# Associations between beverage consumption patterns and adiposity and urinary biomarkers among young adults: a multicenter cross-sectional study in China

**DOI:** 10.3389/fnut.2026.1870358

**Published:** 2026-07-24

**Authors:** Shuyi Zhou, Jianfen Zhang, Yong Jia, Meiling Zhang, Yu Wang, Na Zhang, Guansheng Ma

**Affiliations:** 1 School of Public Health, Peking University, Beijing, China; 2 Department of Student Nutrition, National Institute for Nutrition and Health, Chinese Center for Disease Control and Prevention, Beijing, China; 3 School of Nursing, Jilin University, Changchun, China; 4 School of Public Health, Tianjin Medical University, Tianjin, China; 5 School of Public Health, Lanzhou University, Lanzhou, China; 6 Laboratory of Toxicological Research and Risk Assessment for Food Safety, Peking University, Beijing, China

**Keywords:** adiposity indicators, beverage consumption, fluid intake, plain water, sugar-sweetened beverages, urinary biomarkers, young adults

## Abstract

**Background:**

Beverage consumption is a common dietary behavior that may be associated with adiposity and urinary biomarker profiles, yet population-based evidence in young adults remains limited. This study aimed to examine associations between beverage consumption patterns and adiposity indicators and urinary biomarkers among young adults in China.

**Methods:**

This multicenter cross-sectional study included 3,198 university students aged 18–25 years from seven geographic regions in China. Daily fluid intake (DFI) was assessed using a validated 7-day fluid intake diary with calibrated cups. Body mass index (BMI) and waist circumference (WC) and urinary biomarkers (creatinine, uric acid, uric acid-to-creatinine ratio, and urea) were obtained. Multivariable regression models adjusted for predefined covariates were used to evaluate associations.

**Results:**

Plain water contributed the largest proportion of DFI (83.9%), followed by sugar-sweetened beverages (SSBs), with substantial regional variation in beverage consumption patterns. Higher plain water intake was positively associated with BMI (β = 0.06, 95% CI: 0.02, 0.10, *p* = 0.009) and WC (β = 0.14, 95% CI: 0.02, 0.27, *p* = 0.022), but inversely associated with urinary creatinine (β = −148.05, 95% CI: −197.52, −98.59, *p* < 0.001), urinary uric acid (β = −9.90, 95% CI: −17.58, −2.22, *p* = 0.012), and urinary urea (β = −3.69, 95% CI: −4.74, −2.63, *p* < 0.001). In contrast, SSB intake was positively associated with WC (β = 1.00, 95% CI: 0.62, 1.38, *p* < 0.001), urinary uric acid (β = 45.15, 95% CI: 21.40, 68.89, *p* < 0.001), and urinary uric acid-to-creatinine ratio (*p* < 0.001). Coffee/tea (β = −1.12, 95% CI: −2.18, −0.06, *p* = 0.038), 100% fruit juice (β = −0.86, 95% CI: −1.70, −0.02, *p* = 0.046), and other beverages (β = −1.13, 95% CI: −2.19, −0.07, *p* = 0.037) were inversely associated with waist circumference.

**Conclusion:**

Beverage consumption patterns were associated with adiposity indicators and urinary biomarker profiles among Chinese university students, and these associations differed according to beverage type.

## 1 Introduction

Metabolic diseases, including type 2 diabetes (T2DM), cardiovascular disease (CVD), and chronic kidney disease (CKD), are leading drivers of morbidity, health care costs, and premature mortality worldwide (1). In 2021, 537 million adults were living with T2DM globally, and this number is projected to reach 783 million by 2045 (2). In China, rapid urbanization and industrialization have accelerated dietary transitions characterized by increased consumption of processed foods and sugar-sweetened beverages (SSBs) (3), coinciding with a rising prevalence of obesity, hypertension, and early-onset T2DM (3, 4). Early adulthood represents a critical window for prevention and intervention, as obesity, cardiovascular and metabolic diseases, and CKD often emerge during this life stage (4), potentially influencing future cardiometabolic and kidney-related health outcomes. Understanding modifiable risk factors, such as beverage consumption, during this period is therefore essential for effective, population-level prevention strategies (5).

Different types of beverages have distinct health effects depending on their sugar content and energy density (6). Several studies have supported that higher intake of low-calorie and low sugar intake of beverages such as plain water, unsweetened tea, and coffee have been associated with lower risks of obesity, T2DM, CVD, and specific mortality (7). By contrast, high consumption of SSBs is linked to weight gain, hypertension, T2DM, CKD progression, and premature death (8). However, most of these studies are derived from children, older adults or general population cohorts, and evidence specific to young adults remains limited, despite their distinct lifestyle patterns and ongoing dietary transitions.

Several important research gaps persist. First, most existing studies in China focus on children, older adults or from general population, leaving young adults understudied despite their critical role in primordial prevention (4). Early adulthood is a critical period when cardiometabolic risk factors often first emerge, and modifying behaviors such as beverage consumption during this stage may alter long-term trajectories of T2DM, CVD, and kidney-related health outcomes (9). Second, beverage intake assessment in large-scale studies often relies on 1 to 3 days of 24-h or dietary recalls, which are subject to recall bias and day-to-day variability (10). There is therefore a need for studies using more standardized and extended assessment tools to better capture habitual beverage consumption patterns.

To address these gaps, we conducted a large, multicenter cross-sectional study across seven major geographic regions of China, employing a validated 7-day fluid intake questionnaire with calibrated measuring cups obtain more reliable estimates of habitual beverage consumption. This study aimed to characterize beverage consumption patterns among Chinese young adults and to examine their associations with adiposity indicators, blood pressure, and selected urinary biomarkers. By providing regionally representative evidence on beverage consumption patterns and their associations with adiposity indicators and urinary biomarker profiles, our findings may inform public health strategies and support dietary recommendations targeting young adults in rapidly transitioning settings.

## 2 Methods

### 2.1 Study design

We conducted a multicenter, cross-sectional study between May and June 2023 among university students aged 18–25 years across seven geographic regions of China. To enhance geographic diversity, we applied a multistage stratified sampling design across the seven regions; details of site selection, sampling framework, and sample size calculations are provided in Supplementary methods.

The study protocol was approved by the Ethical Review Committee of Peking University (IRB00001052-22119) and was conducted in accordance with the Declaration of Helsinki. The study protocol was prospectively registered in the Chinese Clinical Trial Registry (ChiCTR2400082286).

### 2.2 Participants

Eligible participants were healthy university students aged 18–25 years. Participants were excluded if they had conditions that could directly alter cardiometabolic or renal physiological status, including a history of chronic diseases, such as renal, metabolic, or cardiovascular diseases, or the use of medications that may affect these outcomes.

Participants were recruited using a combination of online strategies, including WeChat and QQ, and offline strategies, including flyers, booths, and in-class presentations, following a standardized multiregional sampling framework. Detailed recruitment materials, procedures, and consent processes have been described in our previous studies (11, 12).

### 2.3 Assessment of daily fluid intake and beverage classification

Daily fluid intake (DFI) was assessed using the validated 7-day, 24-hour fluid intake diary (Liq.In7), in which participants recorded all fluid consumption by type, volume, and drinking occasion (11–13). DFI was defined as the sum of all these categories. Beverage categories were defined according to the Chinese Beverage Classification Guideline (GB/T 10789-2015) (14), adapted to align with the Chinese Dietary Guidelines and previous epidemiological studies (15): plain water, sugar-sweetened beverages (SSBs), artificially sweetened beverages (ASBs), 100% fruit juices, coffee and tea, alcoholic beverages, and other beverages. Milk and milk derivatives were classified as food water according to the Dietary Guidelines and were excluded from beverage analyses.

### 2.4 Ascertainment of outcomes

Outcomes included metabolic indicators: waist circumference (WC), body mass index (BMI), systolic blood pressure (SBP), and diastolic blood pressure (DBP). Height and weight were measured to the nearest 0.1 cm and 0.1 kg using standardized electronic stadiometers (HDM-300; Huaju, Zhejiang, China) and digital scales (AccuMeasure, Greenwood Village, CO, USA). BMI was calculated as weight (kg) divided by height squared (m^2^). WC was measured at the midpoint between the lowest rib and the iliac crest using a non-stretchable tape with 0.1 cm precision. Blood pressure was measured twice with an automated sphygmomanometer (Omron HEM-7200; Omron Healthcare, Kyoto, Japan) after a 5-min rest in the seated position, and the mean of the two readings was used for analysis.

Renal biomarkers included urinary creatinine (Cr), urinary uric acid (UA), urinary urea, and the urinary uric acid-to-creatinine ratio (UA/Cr). All renal biomarkers were measured using 24-h urine samples collected from participants. Urine samples were centrifuged and analyzed using an automated biochemical analyzer (Beckman Coulter AU2700, Beckman Coulter, USA). Urinary urea was measured using the urease–glutamate dehydrogenase method, urinary creatinine using the Jaffe method, and urinary uric acid using the uricase colorimetric method. The UA/Cr ratio was calculated as urinary uric acid divided by urinary creatinine.

### 2.5 Ascertainment of covariates

Covariates were selected *a priori* based on theoretical relevance and previous literature. Demographic covariates included age, sex, ethnicity, and socioeconomic status, with cities classified into first-tier, new first-tier, second-tier cities, and third-tier cities based on the Chinese Tier City System (16). This classification is based on economic development, transportation system, infrastructure and cultural significance in China according to data from National Bureau of Statistics of the People's Republic of China.

Lifestyle variables included physical activity assessed by the International Physical Activity Questionnaire (IPAQ) (17). Dietary variables included total daily energy intake, sodium intake, and macronutrient distribution (protein, carbohydrate, and fat). Psychological status was evaluated using the Self-Rating Anxiety Scale (SAS) (18) and the Self-Rating Depression Scale (SDS) (19).

Data collection followed standardized procedures, including investigator-administered questionnaires, duplicate portion dietary validation, 24-h urine-based sodium estimation, and routine monitoring of environmental conditions. Detailed methods, including sample handling, nutrient analysis standards, and the formula for sodium calculation, are provided in Supplementary Methods.

Rigorous quality control procedures, centralized training, double data entry, and anonymized data management were applied throughout the study. Detailed quality assurance measures are provided in the Supplementary Methods.

### 2.6 Statistics analysis

Descriptive statistics were used to summarize sample characteristics. The distribution of continuous variables was assessed using the Kolmogorov–Smirnov test, and all variables were found to be non-normally distributed; therefore, they are presented as medians with interquartile ranges (IQRs). Group comparisons were performed using the Mann–Whitney U test when comparing two groups and the Kruskal–Wallis test when comparing more than two groups. Where the Kruskal–Wallis test was significant, *post-hoc* comparisons were conducted using Dunn's test with Bonferroni correction. Categorical variables were expressed as absolute frequencies and percentages, and compared using the χ^2^ test or Fisher's exact test, as appropriate. Missing values were not imputed, and all analyses were conducted using complete-case data.

Covariates were selected *a priori* based on existing literature, biological plausibility, and their potential associations with both beverage consumption and health outcomes (12, 20, 21). These included demographic and socioeconomic factors (age, sex, ethnicity, city tier classification, geographic region), lifestyle factors (physical activity from IPAQ), psychological status (SAS and SDS scores), and dietary factors (total energy intake, sodium intake, and macronutrient categories) (21). Macronutrient thresholds were defined according to Chinese dietary reference standards: high protein (>65 g/day for men, >55 g/day for women), high carbohydrate (>150 g/day), and high fat (>30% of total energy intake) (15). To account for the multicenter study design and potential clustering of participants within geographic regions, geographic region was included as a random intercept in all mixed-effects regression models. Beverage intake variables and all covariates were specified as fixed effects.

To assess potential multicollinearity among covariates, variance inflation factors (VIFs) were calculated for the fully adjusted models. Sensitivity analyses were performed by refitting the models without adjustment for total energy intake. Additional sensitivity analyses were conducted to evaluate the robustness of the observed associations between plain water intake and adiposity indicators, including: (1) exclusion of underweight and obese participants; (2) sex-stratified analyses; (3) substitution of DFI for plain water intake as the exposure variable; and (4) robust mixed-effects regression to account for potential influential outliers. Furthermore, sex-specific interaction analyses were performed by including interaction terms between beverage intake variables and sex in the fully adjusted models. For statistically significant interactions, sex-stratified analyses were conducted to further examine the associations within each sex.

## 3 Results

### 3.1 Characteristics of participants

The recruitment process is shown in Supplementary Figure 1, and the characteristics of participants are summarized in Table 1. A total of 3,198 young adults (median age 20 years; IQR: 19–20) were included in this multicenter cross-sectional study. Among them, 1,748 (54.9%) were female and 1,437 (45.1%) were male. Of these, 2,817 (88.1%) were of Han ethnicity. Participants were recruited from seven geographic regions across China: Northeast (*n* = 499, 15.6%), North (*n* = 316, 9.9%), Northwest (*n* = 502, 15.7%), East (*n* = 398, 12.4%), Central (*n* = 498, 15.6%), Southwest (*n* = 466, 14.6%), and South (*n* = 519, 16.2%).

**Table 1:** Baseline characteristics of study participants by sex.

Variable	Overall (*N* = 3,198)
Demographic characteristics	
Age (years)	20.0 (19.0, 20.0)
Sex	
Female	1,748 (54.7%)
Male	1,443 (45.1%)
Ethnicity, *n* (%)	
Han	2,817 (88.1%)
Minority	361 (11.3%)
Region	
Northeast China (Changchun, CC)	499 (15.6%)
North China (Tianjin, TJ)	316 (9.9%)
Northwest China (Lanzhou, LZ)	502 (15.7%)
East China (Shanghai, SH)	398 (12.4%)
Central China (Changsha, CS)	498 (15.6%)
Southwest China (Yunnan, YN)	466 (14.6%)
South China (Haikou, HK)	519 (16.2%)
Lifestyle factors	
Physical activity level, *n* (%)	
Low	552 (17.3%)
Medium	1,322 (41.3%)
High	1,294 (40.5%)
Dietary intake	
^†^Daily energy intake (kcal/day)	1,715 (1,410, 2,067)
^†^Daily fat intake (g/day)	71 (59, 85)
^†^Daily protein intake (g/day)	60 (50, 73)
^†^Daily carbohydrate intake (g/day)	226 (179, 285)
^†^Daily salt intake (g/day)	6.9 (5.0, 9.6)
Psychological factors	
SAS scale	35 (32, 39)
SDS index	42 (38, 46)
Water intake variables	
DFI (mL/day)	1,280 (939, 1,593)
Health outcomes	
BMI category, *n* (%)	
Underweight	509 (15.9%)
Normal weight	2,016 (63.0%)
Overweight	526 (16.4%)
Obesity	122 (3.8%)
Waist circumference (WC, cm)	72 (67, 80)
SBP (mmHg)	115 (106, 121)
DBP (mmHg)	73 (67, 78)
^†^Creatinine (Cr, μmol/L)	8,387 (5,889, 11,699)
^†^Uric acid (UA, μmol/L)	1,214 (820, 1,721)
^†^UA/Cr ratio	0.19 (0.10, 0.29)
^†^Urea (μmol/L)	218 (157, 294)

Data are presented as median (IQR) for continuous variables and *n* (%) for categorical variables. Sample sizes differ by sex due to missing data (*N* = 13). Group differences were assessed using the Mann–Whitney U test for continuous variables and the χ^2^ test for categorical variables. †Variables are based on a subsample (*n* = 945). CC, Changchun; TJ, Tianjin; LZ, Lanzhou; SH, Shanghai; CS, Changsha; YN, Yunnan; HK, Haikou; DFI, drinking fluid intake; TWI, total water intake; WC, waist circumference; SBP, systolic blood pressure; DBP, diastolic blood pressure; SAS, Self-Rating Anxiety Scale; SDS, Self-Rating Depression Scale; UA/Cr, uric acid-to-creatinine ratio.

Regarding lifestyle characteristics, 552 (17.3%) reported low physical activity, 1,322 (41.3%) moderate, and 1,294 (40.5%) high. The median daily energy intake was 1,715 kcal (IQR 1,410–2,067), with intakes of 71 g fat (IQR 59–85), 60 g protein (IQR 50–73), 226 g carbohydrates (IQR 179–285), and 6.9 g salt (IQR 5.0–9.6). The median DFI was 1,280 mL (IQR 939–1,593).

Anthropometric parameters showed that 509 (15.9%) were underweight, 2,016 (63.0%) had normal weight, 526 (16.4%) were overweight, and 122 (3.8%) were obese. The median WC was 72 cm (IQR 67–80), with median SBP and DBP of 115 mmHg (IQR 106–121) and 73 mmHg (IQR 67–78), respectively. Median urinary creatinine, uric acid, uric acid-to-creatinine ratio, and urea were within normal ranges.

### 3.2 Beverage consumption patterns

Within DFI, plain water constituted the largest volume, with a median intake of 1,050 mL (IQR 750–1,357), followed by SSBs (50 mL, IQR 0–150). Intakes of coffee and tea, 100% fruit juice, ASBs, and alcoholic beverages were generally low, with median values of 0 mL across categories (Table 2). Significant sex differences were observed in several beverage categories. Compared with women, men reported higher median intakes of plain water (1,108 vs. 1,000 mL; *p* < 0.001) and SSBs (52 vs. 47 mL; *p* = 0.049). Although median intakes of coffee and tea were 0 mL in both groups, distributions differed significantly (*p* < 0.001).

**Table 2:** Drinking fluid intake by overall and sex.

Beverage	Overall (*N* = 3,198)	Male (*N* = 1,450)	Female (*N* = 1,748)	*p* value
Water	1,050 (750–1357)	1,108 (836–1,419)	1000 (677–1,296)	< 0.001
Coffee/tea	0 (0–50)	0 (0–36)	0 (0–57)	< 0.001
100% fruit juices	0 (0–0)	0 (0–0)	0 (0–0)	< 0.001
SSB	50 (0–150)	52.46 (0–171)	46.87 (0–143)	0.049
Other beverages	0 (0–50)	0 (0–53.07)	0 (0–43)	0.489

Data are presented as median (IQR, mL/day). Sample sizes differ slightly by sex due to missing data (*n* = 13). *P* values were calculated using the Mann–Whitney U test. Artificially sweetened beverages (ASBs) and alcoholic beverages are not shown due to median intakes of 0 mL across groups; however, statistically significant differences reflect variations in distribution rather than central tendency.

**Figure 1:**
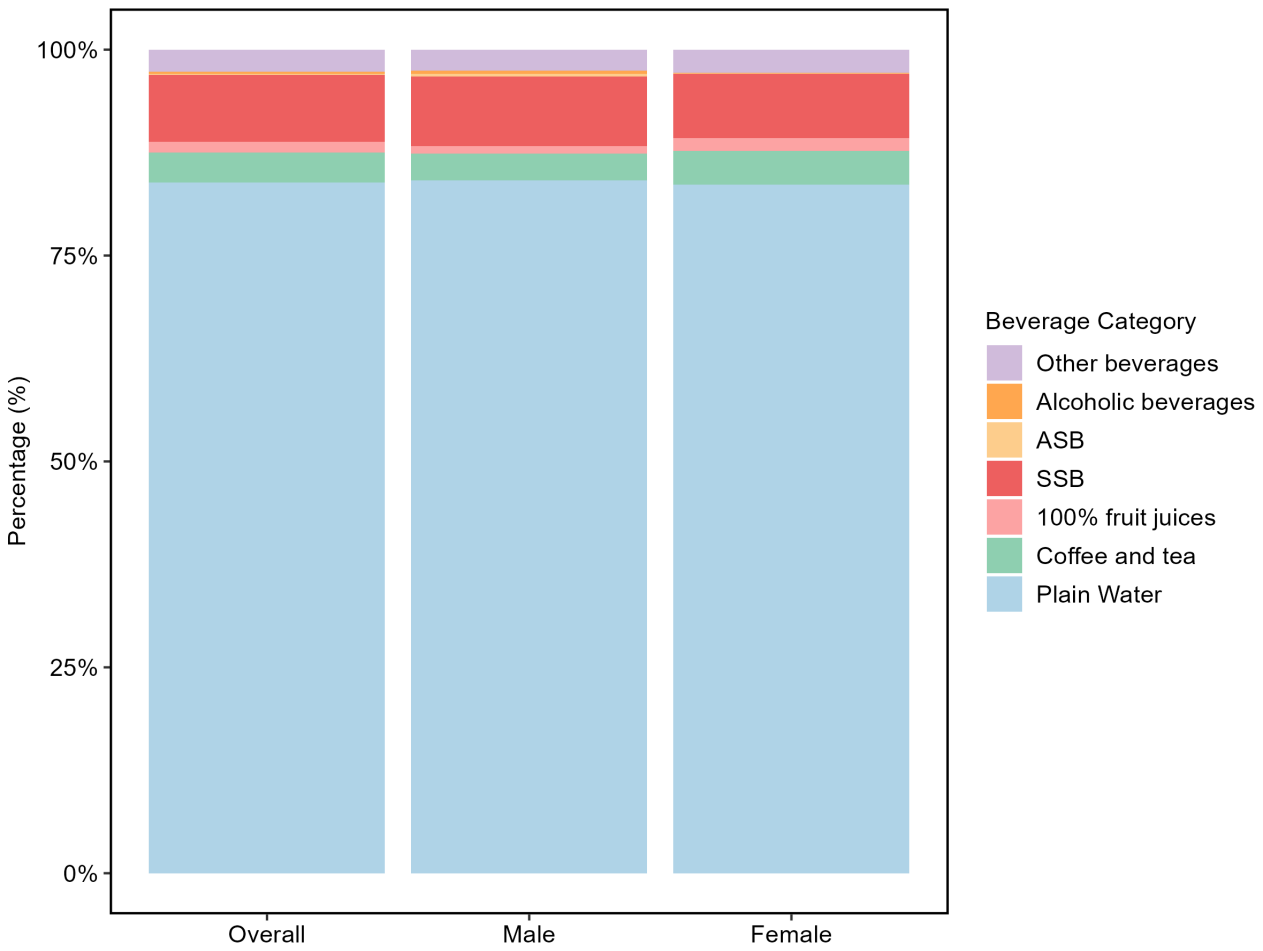
Distribution of daily drinking fluid intake by beverage category and gender. This stacked bar chart illustrates the percentage contribution of beverage categories to total daily drinking fluid intake across the overall population and by sex (male, female). Colors represent beverage categories as follows: light blue, plain water; light green, coffee and tea; light pink, 100% fruit juices; red, sugar-sweetened beverages (SSB); pale yellow, artificially sweetened beverages (ASB); orange, alcoholic beverages; purple, other beverages. The y-axis shows the proportion (%) of each beverage category within total daily fluid intake, facilitating comparison across groups.

Proportional contribution analysis showed that plain water accounted for 83.9% of DFI, followed by SSBs (8.1%), coffee and tea (3.7%), and other beverages (2.7%). The contributions of 100% fruit juices, ASBs, and alcoholic beverages were each below 2% (Figure 1). Sex-specific patterns were generally consistent across beverage categories. Compared with women, men had a higher proportional contribution of SSBs, whereas women showed relatively greater contributions from coffee/tea and fruit juices.

### 3.3 Regional disparities in beverage consumption patterns

Significant sex- and region-based variations were observed in beverage consumption patterns (*p* < 0.001) (Supplementary Table 1). Plain water accounted for the largest proportion of DFI across all regions, ranging from 76.2% in East China (SH) to 88.7% in South China (HK). East China (SH) had the highest proportion of SSBs (12.5%), while Southwest China (YN) had the largest contribution from fruit juices (5.1%). The proportion of coffee and tea intake was highest in SH (5.7%) and lowest in YN (1.0%). ASBs contributed minimally to DFI.

Regional differences in beverage intake are presented in Supplementary Table 2. The median daily intake of plain water ranged from 857 mL (IQR 551–1,146) in East China (SH) to 1,272 mL (IQR 1,117–1,423) in South China (HK). SSBs consumption was highest in Central China (CS; 107 mL, IQR 29–229; *p* < 0.001), while intake was minimal in Southwest China (YN). Coffee and tea consumption showed a similar regional pattern, with higher intake in Central China (CS) and minimal intake in Southwest China (YN) (*p* < 0.001). The intake of 100% fruit juices, ASBs, alcoholic beverages, and other beverages was generally low across all regions, although statistically significant regional differences were observed (*p* < 0.001).

**Figure 2:**
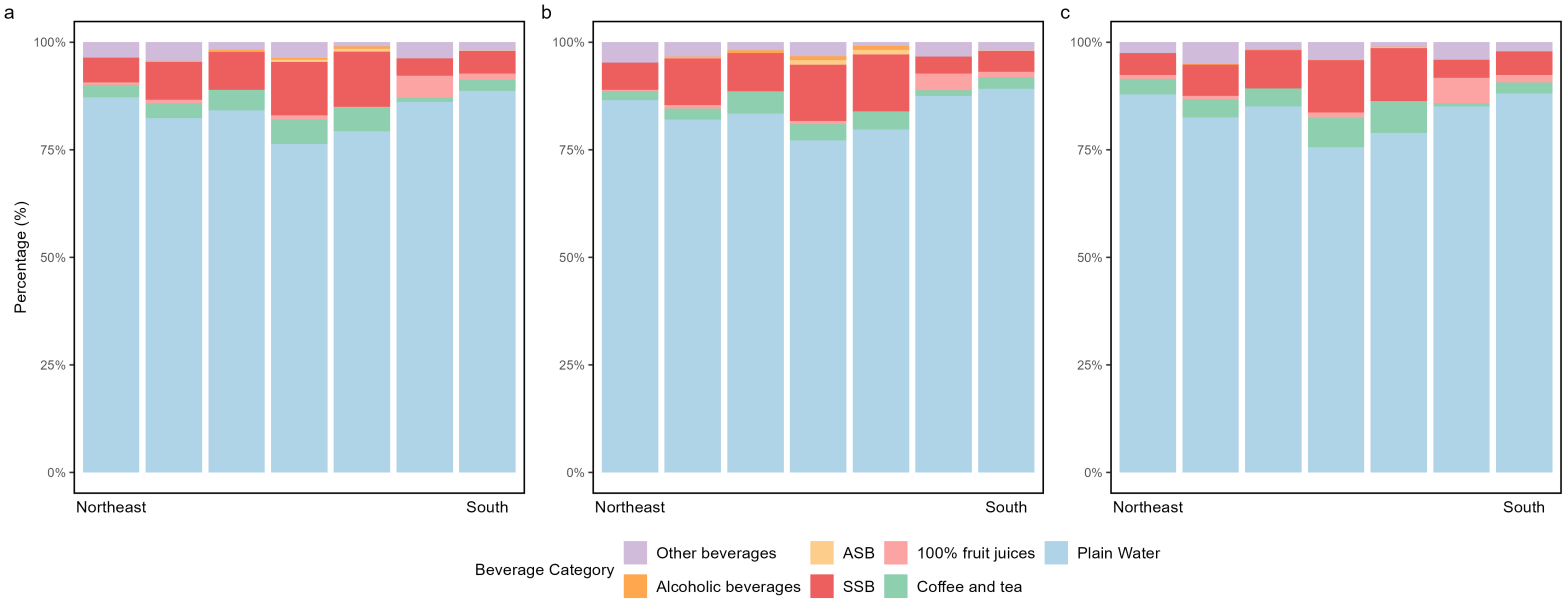
Distribution of beverage categories contributing to daily fluid intake by region and sex. Stacked bar charts show the proportional contribution (%) of beverage categories to total daily fluid intake across seven regions of China (from Northeast to South) in the overall population **(a)**, males **(b)**, and females **(c)**. Beverage categories include plain water, coffee and tea, 100% fruit juices, sugar-sweetened beverages (SSBs), artificially sweetened beverages (ASBs), alcoholic beverages, and other beverages.

Sex-specific differences were observed within regions (Figure 2). Men had a higher proportional contribution of SSBs, whereas women showed relatively greater contributions from coffee/tea and fruit juices. For example, in East China (SH), the contribution of SSBs was higher in men than in women (13.0% vs. 12.1%), while coffee and tea accounted for a greater proportion of DFI in women than in men (6.8% vs. 3.8%). Similar patterns were observed in Central China.

### 3.4 Associations of different beverage types with health outcomes

Associations between beverage intake and health outcomes were examined using multivariable regression models adjusted for predefined covariates. Health outcomes assessed included adiposity measures (BMI and waist circumference), blood pressure parameters (SBP and DBP), and urinary biomarkers (urinary creatinine, urinary uric acid, urinary uric acid-to-creatinine ratio, and urinary urea) (Table 3).

**Table 3A:** Associations between beverage intake (per 100 mL increase) and adiposity indicators.

Beverage Types	BMI (kg/m^2^)		WC (cm)		SBP (mmHg)		DBP (mmHg)	
	β [95% CI]	*p*	β [95% CI]	*p*	β [95% CI]	*p*	β [95% CI]	*p*
Plain Water	0.06 [0.02, 0.10]	0.009	0.14 [0.02, 0.27]	0.022	0.09 [−0.04, 0.22]	0.155	0.01 [−0.09, 0.11]	0.825
Coffee/Tea	−0.28 [−0.65, 0.08]	0.128	−1.12 [−2.18, −0.06]	0.038	−0.37 [−1.48, 0.74]	0.512	−0.33 [−1.16, 0.50]	0.433
Fruit Juices	−0.27 [−0.55, 0.02]	0.068	−0.86 [−1.70, −0.02]	0.046	0.00 [−0.88, 0.89]	0.995	−0.50 [−1.16, 0.16]	0.137
SSB	0.11 [−0.02, 0.24]	0.098	1.00 [0.62, 1.38]	< 0.001	0.27 [−0.13, 0.67]	0.188	0.27 [−0.02, 0.57]	0.071
ASB	1.19 [0.28, 2.10]	0.010	5.95 [3.38, 8.51]	< 0.001	3.27 [0.58, 5.97]	0.017	0.92 [−1.09, 2.94]	0.369
Alcoholic	−0.30 [−1.27, 0.68]	0.550	−0.77 [−3.55, 2.00]	0.585	1.84 [−1.05, 4.73]	0.211	0.70 [−1.46, 2.85]	0.526
Other Beverages	−0.28 [−0.65, 0.08]	0.126	−1.13 [−2.19, −0.07]	0.037	−0.37 [−1.48, 0.74]	0.512	−0.33 [−1.16, 0.50]	0.432

β (95% CI) represent changes in outcomes per 100 mL/day increase in beverage intake. Models were adjusted for predefined covariates, with geographic region included as a random intercept. *p* values were derived from mixed-effects models.

**Table 3B:** Associations between beverage intake (per 100 mL increase) and urinary biomarkers.

Beverage Types	Urinary Cr (μmol/L)		Urinary UA (μmol/L)		Urinary UA/Cr		Urinary urea (μmol/L)	
	β [95% CI]	*p*	β [95% CI]	*p*	β [95% CI]	*p*	β [95% CI]	*p*
Plain Water	−148.05 [−197.52, −98.59]	< 0.001	−9.90 [−17.58, −2.22]	0.012	0.00 [0.00, 0.00]	0.011	−3.69 [−4.74, −2.63]	< 0.001
Coffee/Tea	185.34 [−248.69, 619.37]	0.402	−7.00 [−73.55, 59.54]	0.836	−0.01 [−0.03, 0.01]	0.230	1.98 [−7.36, 11.31]	0.677
Fruit Juices	15.59 [−331.06, 362.24]	0.930	0.22 [−53.01, 53.44]	0.994	−0.00 [−0.01, 0.01]	0.969	−2.08 [−9.52, 5.37]	0.584
SSB	−27.59 [−183.95, 128.77]	0.729	45.15 [21.40, 68.89]	< 0.001	0.01 [0.01, 0.02]	< 0.001	−1.72 [−5.08, 1.64]	0.316
ASB	502.79 [−563.48, 1569.06]	0.355	−38.25 [-201.18, 124.67]	0.645	−0.00 [−0.05, 0.04]	0.939	8.09 [−14.89, 31.08]	0.490
Alcoholic	−227.69 [−1,367.62, 912.23]	0.695	15.48 [−158.71, 189.66]	0.862	0.00 [−0.04, 0.05]	0.879	1.49 [−23.07, 26.05]	0.905
Other Beverages	185.25 [−249.05, 619.56]	0.403	−7.19 [−73.77, 59.38]	0.832	−0.01 [-0.03, 0.01]	0.225	2.06 [−7.28, 11.39]	0.665

All renal biomarkers presented in this table were derived from 24-h urine specimens. β (95% CI) represent changes in outcomes per 100 mL/day increase in beverage intake. Models were adjusted for predefined covariates, with geographic region included as a random intercept. *p* values were derived from mixed-effects models.

Plain water intake was positively associated with BMI (β = 0.06, 95% CI: 0.02, 0.10, *p* = 0.009) and WC (β = 0.14, 95% CI: 0.02, 0.27, *p* = 0.022). Coffee/tea intake was inversely associated with WC (β = −1.12, 95% CI: −2.18, −0.06, *p* = 0.038). Similarly, 100% fruit juice intake (β = −0.86, 95% CI: −1.70, −0.02, *p* = 0.046) and other beverages intake (β = −1.13, 95% CI: −2.19, −0.07, *p* = 0.037) were inversely associated with WC. SSB intake was positively associated with WC (β = 1.00, 95% CI: 0.62, 1.38, *p* < 0.001). ASB intake was positively associated with BMI (β = 1.19, 95% CI: 0.28, 2.10, *p* = 0.010), WC (β = 5.95, 95% CI: 3.38, 8.51, *p* < 0.001), and SBP (β = 3.27, 95% CI: 0.58, 5.97, *p* = 0.017).

For urinary biomarkers, plain water intake was associated with lower urinary creatinine (β = −148.05, 95% CI: −197.52, −98.59, *p* < 0.001), urinary uric acid (β = −9.90, 95% CI: −17.58, −2.22, *p* = 0.012), and urinary urea (β = −3.69, 95% CI: −4.74, −2.63, *p* < 0.001), and with a higher urinary uric acid-to-creatinine ratio (*p* = 0.011). SSB intake was associated with higher urinary uric acid (β = 45.15, 95% CI: 21.40, 68.89, *p* < 0.001) and urinary uric acid-to-creatinine ratio (*p* < 0.001).

### 3.5 Sensitivity analyses

All VIF values ranged from 1.01 to 2.06, indicating no evidence of substantial multicollinearity among the fixed-effect covariates (Supplementary Table 3). Sensitivity analyses excluding total energy intake yielded results similar to those of the primary analyses (Supplementary Table 4).

Additional sensitivity analyses evaluating the positive association between plain water intake and adiposity indicators are presented in Supplementary Table 5. The association with BMI remained statistically significant after excluding underweight and obese participants, substituting DFI for plain water intake, and applying robust regression models. For WC, positive associations were observed across all sensitivity analyses, although statistical significance was not retained after excluding underweight and obese participants or in sex-stratified analyses.

### 3.6 Sex-specific interaction analyses

Sex-specific interaction analyses are presented in Supplementary Table 6. No significant interactions were observed between beverage consumption and anthropometric or blood pressure outcomes (all *p* > 0.05; Supplementary Table 6A). For urinary biomarkers, significant sex-specific interactions were observed between plain water intake and creatinine (*p* = 0.036), uric acid-to-creatinine ratio (*p* = 0.025), and urea (*p* = 0.005) (Supplementary Table 6B). Detailed sex-stratified results are provided in Supplementary Table 6B. No significant interactions were observed for other beverage types.

## 4 Discussion

### 4.1 Principle findings

This multicenter study of young adults from seven geographic regions of China shows that plain water remains the dominant source of beverages among young Chinese adults, while SSBs account for a relatively small but important share. Men consumed more SSBs than women, and pronounced geographic heterogeneity was observed, with intake highest in Eastern and Central China and lowest in the Southwest. Higher SSB consumption was associated with greater WC. In contrast, higher intakes of coffee, tea, and 100% fruit juice were inversely associated with WC, while plain water intake was associated with lower urinary creatinine, uric acid, and urea concentrations.

### 4.2 Comparison with other studies and possible explanations

Our findings are consistent with reports from other Asian cohorts, which also describe water-dominant beverage consumption patterns (21), whereas Western populations typically derive nearly 40–80% of beverage energy from SSBs (5). This contrast highlights the role of sociocultural and environmental contexts in shaping beverage behaviors, which may contribute to differences in population-level dietary exposures and related health outcomes. Nonetheless, national surveillance data in China have documented a steady rise in SSB consumption over the past two decades, with the most rapid increase among adolescents and young adults (22). If this upward trajectory continues, the public health relevance of SSB consumption among young adults may increase further (23).

Regional disparities in SSB consumption in China reflect a complex interplay of urbanization, socioeconomic factors, local food environments, cultural preferences, and climate conditions (24, 25). Higher SSB intake in Eastern (SH) and Central China (CS) is associated with greater urbanization (25), higher socioeconomic status (25), and denser retail networks (26), including convenience stores and bubble tea shops, which increase beverage accessibility and marketing exposure (26). These factors align with broader evidence linking urban dietary transitions to increased availability of energy-dense, nutrient-poor beverages. In addition, warmer and more humid conditions in these regions are associated with higher SSB consumption (24). In contrast, Southern China (HK), despite similar hot and humid conditions, exhibits relatively lower SSB intake, which may partly reflect a greater consumption of coconut water, classified as 100% fruit juice in the present study. Southwest China (YN) also demonstrates lower consumption, consistent with lower urbanization levels and more traditional water-drinking habits (27). These pronounced geographic variations highlight the need to consider regional context when interpreting beverage consumption patterns among young adults.

The observed sex differences, with men consuming more SSBs, are consistent with national and global nutrition surveys (5, 22). Potential explanations include higher energy requirements and differing beverage preferences among men, as well as gender-specific attitudes toward body weight and health behaviors. Sociocultural norms and body image concerns may partly explain lower intake among women; however, the underlying mechanisms remain unclear and warrant further investigation. The coexistence of sex and regional disparities highlights potential inequalities in exposure to unhealthy dietary environments and related metabolic outcomes. The coexistence of sex and regional disparities highlights differences in beverage consumption behaviors and potential exposure to unhealthy dietary environments. Given the cross-sectional design, causal inferences cannot be established, and future longitudinal studies incorporating biomarkers are needed to clarify the pathways linking beverage patterns to metabolic outcomes. These findings may provide evidence to support future efforts to promote healthy beverage consumption among young adults in China.

### 4.3 Potential explanations

The association between higher SSB intake and central adiposity may be explained by several biological pathways. SSBs substantially increase dietary free sugar and energy intake, with limited satiety compensation compared with solid foods, which has been associated with positive energy balance in previous studies (28). Excess sugar intake has been linked to disruptions in metabolic homeostasis, including rapid glycaemic and insulinaemic responses, insulin resistance, hyperuricaemia, dyslipidaemia, and chronic low-grade inflammation, which have been associated with increased cardiometabolic risk (28).

The observed association of SSB intake with WC, but not BMI, may reflect differences in the adiposity measures captured by these indicators. WC is a more sensitive indicator of abdominal and visceral adiposity than BMI. Previous studies have suggested that fructose, a major component of many SSBs, may be associated with preferential visceral fat accumulation through hepatic *de novo* lipogenesis and insulin resistance, which could partly explain the stronger association observed for waist circumference than for BMI (29–31).

Higher plain water intake was positively associated with BMI and WC in the present study. Similar associations have been reported previously and may reflect greater physiological water requirements among individuals with larger body size and higher metabolic demands (32, 33). Sensitivity analyses generally supported the association with BMI, whereas findings for WC were less consistent across alternative model specifications. Given the cross-sectional design and the relatively small effect sizes observed, the direction and clinical significance of these associations remain uncertain. Higher plain water intake was also associated with lower urinary creatinine, uric acid, and urea concentrations. These associations may partly reflect physiological processes such as greater urinary dilution and lower urine solute concentrations (34).

Coffee/tea and 100% fruit juice were inversely associated with waist circumference, consistent with findings from previous studies. Bioactive compounds such as chlorogenic acids in coffee and catechins in tea have been linked to more favorable metabolic outcomes in observational and intervention studies, while moderate consumption of 100% fruit juice has been associated with higher overall diet quality and more favorable nutrient intake patterns (35).

### 4.4 Strengths and limitations of this study

This multicenter study spanning seven geographic regions of China provides comprehensive data on beverage consumption patterns among healthy young adults. The use of a validated seven-day fluid intake questionnaire together with calibrated measuring containers helped improve the accuracy of beverage intake assessment. In addition, the standardized multicenter protocol enhanced comparability across the seven geographic regions.

However, several limitations warrant consideration. First, the cross-sectional design precludes causal inference. Second, all participants were university students, which may limit the generalizability of the findings to non-student young adults and other population groups (e.g., rural youth and migrant workers) in China. Third, beverage intake was self-reported and therefore subject to potential reporting bias. Fourth, residual confounding cannot be excluded despite adjustment for multiple covariates. Future research should adopt longitudinal designs and incorporate repeated dietary assessments, objective biomarkers, and digital intake monitoring to better clarify the long-term health implications of beverage consumption patterns.

## 5 Conclusion

In this multicenter study across seven geographic regions of China, plain water remained the predominant beverage type among university students aged 18–25 years, although substantial sex and regional differences in beverage consumption patterns were observed. Higher SSB intake was associated with greater waist circumference, whereas higher plain water intake was associated with lower urinary creatinine, uric acid, and urea concentrations. These findings provide multiregional evidence on beverage consumption patterns and their associations with anthropometric and urinary biomarkers among Chinese university students.

## Data Availability

The raw data supporting the conclusions of this article will be made available by the corresponding author upon reasonable request.

## References

[1] Network GBoDC. Global Burden of Disease (GBD) 2000–2019. (2019). Available online at: https://vizhub.healthdata.org/gbd-results/ (Accessed July 11, 2026).

[2] Sun H Saeedi P Karuranga S Pinkepank M Ogurtsova K Duncan BB . IDF diabetes atlas: global, regional and country-level diabetes prevalence estimates for 2021 and projections for 2045. Diabetes Res Clin Pract. (2022) 183:109119. doi: 10.1016/j.diabres.2021.109119 34879977 PMC11057359

[3] Pan XF Wang L Pan A. Epidemiology and determinants of obesity in China. Lancet Diabetes Endocrinol. (2021) 9:373–92. doi: 10.1016/S2213-8587(21)00045-0 34022156

[4] Leunissen RW Kerkhof GF Stijnen T Hokken-Koelega A. Timing and tempo of first-year rapid growth in relation to cardiovascular and metabolic risk profile in early adulthood. JAMA. (2009) 301:2234–42. doi: 10.1001/jama.2009.761 19491185

[5] Popkin BM Hawkes C. Sweetening of the global diet, particularly beverages: patterns, trends, and policy responses. Lancet Diabetes Endocrinol. (2016) 4:174–86. doi: 10.1016/S2213-8587(15)00419-2 26654575 PMC4733620

[6] de Oliveira Otto MC Anderson CAM Dearborn JL Ferranti EP Mozaffarian D. Dietary diversity: implications for obesity prevention in adult populations: a science advisory from the American Heart Association. Circulation. (2018) 138:e160–8. doi: 10.1161/CIR.0000000000000595 30354383 PMC6894732

[7] Di Maso M Boffetta P Negri E La Vecchia C Bravi F. Caffeinated coffee consumption and health outcomes in the US population: a dose-response meta-analysis and estimation of disease cases and deaths avoided. Adv Nutr. (2021) 12:1160–76. doi: 10.1093/advances/nmaa177 33570108 PMC8321867

[8] Ma L Hu Y Alperet DJ Liu G Malik V Manson JE . Beverage consumption and mortality among adults with type 2 diabetes: prospective cohort study. BMJ. (2023) 381:e073406. doi: 10.1136/bmj-2022-073406 37076174 PMC10114037

[9] Scott J Agarwala A Baker-Smith CM Feinstein MJ Jakubowski K Kaar J . Cardiovascular health in the transition from adolescence to emerging adulthood: a scientific statement from the American Heart Association. J Am Heart Assoc. (2025) 14:e039239. doi: 10.1161/JAHA.124.039239 40135400 PMC12184556

[10] Morin C Gandy J Moreno LA Kavouras SA Martinez H Salas-Salvadó J . A comparison of drinking behavior using a harmonized methodology (Liq.In^7^) in six countries. Eur J Nutr. (2018) 57:101–12. doi: 10.1007/s00394-018-1744-8 29923118 PMC6008358

[11] Zhou S Zhang J Shen X Wang Y Zhang M Jia Y . Water from food in young Chinese adults: patterns, determinants, and public health implications: a cross-sectional study across the seven geographic regions. Foods. (2025) 15:29. doi: 10.3390/foods15010029 41517097 PMC12785264

[12] Zhou S Zhang J Shen X Yang L He J Zhang F . Determinants of beverage consumption in young adults: a multicenter cross-sectional study across seven major geographic regions of China. Foods. (2025) 14:3687. doi: 10.3390/foods14213687 41227659 PMC12607530

[13] Zhang N Zhang J Ma GS. Development, validation, and application of the 7-day 24-hour drinking water survey questionnaire. Acta Nutr Sin. (2024) 46:177–82+91.

[14] General Administration of Standardization. General Rules for Beverage. Beijing: General Administration of Standardization (2015).

[15] Chinese Nutrition Society. Chinese Dietary Guidelines (2022). Beijing: People's Medical Publishing House (2022).

[16] State Council of China. Notice of the State Council on Adjusting the Criteria for Classifying the Size of Cities. Beijing: State Council of China (2014).

[17] Ekingen T Sob C Hartmann C Rühli FJ Matthes KL Staub K . Associations between hydration status, body composition, sociodemographic and lifestyle factors in the general population: a cross-sectional study. BMC Public Health. (2022) 22:900. doi: 10.1186/s12889-022-13280-z 35513819 PMC9071243

[18] Zung WW. A rating instrument for anxiety disorders. Psychosomatics. (1971) 12:371–9. doi: 10.1016/S0033-3182(71)71479-0 5172928

[19] Zung WW. A self-rating depression scale. Arch Gen Psychiatry. (1965) 12:63–70. doi: 10.1001/archpsyc.1965.01720310065008 14221692

[20] Maughan RJ Watson P Cordery PA Walsh NP Oliver SJ Dolci A . A randomized trial to assess the potential of different beverages to affect hydration status: development of a beverage hydration index. Am J Clin Nutr. (2016) 103:717–23. doi: 10.3945/ajcn.115.114769 26702122

[21] Zhang N Morin C Guelinckx I Moreno LA Kavouras SA Gandy J . Fluid intake in urban China: results of the 2016 Liq.In^7^ national cross-sectional surveys. Eur J Nutr. (2018) 57:77–88. doi: 10.1007/s00394-018-1755-5 29923120 PMC6008349

[22] Pan F Li D Zhang TW Mao WF Liang D Liu AD . Consumption status of sugar-sweetened beverages and free sugar intake among urban residents aged 3 years and above in China. Chin J Food Hyg. (2022) 34:126–30. doi: 10.13590/j.cjfh.2022.01.024

[23] Central Central Committee of the Communist Party of China State Council. Healthy China Action (2019–2030). (2016). Available online at: https://www.gov.cn/ (Accessed October 25, 2016).

[24] He P Xu Z Chan D Liu P Bai Y. Rising temperatures increase added sugar intake disproportionately in disadvantaged groups in the USA. Nat Clim Change. (2025) 15:963–70. doi: 10.1038/s41558-025-02398-8

[25] Guo H Phung D Chu C. Sociodemographic, lifestyle, behavioral, and parental factors associated with sugar-sweetened beverage consumption in children in China. PLoS ONE. (2021) 16:e0261199. doi: 10.1371/journal.pone.0261199 34890424 PMC8664181

[26] Paes VM Hesketh K O'Malley C Moore H Summerbell C Griffin S. Determinants of sugar-sweetened beverage consumption in young children: a systematic review. Obes Rev. (2015) 16:903–13. doi: 10.1111/obr.12310 26252417 PMC4737242

[27] Yao K Shuping Y. Study on spatial distribution of modern sweet diet and its impact factors in China based on big data from internet. J Geo-Inf Sci. (2020) 22:1202–15. doi: 10.12082/dqxxkx.2020.190432

[28] Malik VS Popkin BM Bray GA Després JP Hu FB. Sugar-sweetened beverages, obesity, type 2 diabetes mellitus, and cardiovascular disease risk. Circulation. (2010) 121:1356–64. doi: 10.1161/CIRCULATIONAHA.109.876185 20308626 PMC2862465

[29] Ma J McKeown NM Hwang SJ Hoffmann U Jacques PF Fox CS. Sugar-sweetened beverage consumption is associated with change of visceral adipose tissue over 6 years of follow-up. Circulation. (2016) 133:370–7. doi: 10.1161/CIRCULATIONAHA.115.018704 26755505 PMC4729662

[30] Malik VS Hu FB. The role of sugar-sweetened beverages in the global epidemics of obesity and chronic diseases. Nat Rev Endocrinol. (2022) 18:205–18. doi: 10.1038/s41574-021-00627-6 35064240 PMC8778490

[31] Stanhope KL Schwarz JM Keim NL Griffen SC Bremer AA Graham JL . Consuming fructose-sweetened, not glucose-sweetened, beverages increases visceral adiposity and lipids and decreases insulin sensitivity in overweight/obese humans. J Clin Invest. (2009) 119:1322–34. doi: 10.1172/JCI37385 19381015 PMC2673878

[32] Abdulsalam R Alsadah A Alkhuboli M Muala D Hussein A Elmoselhi AB. Hydration status assessment and impinging factors among university students in the UAE. Eur Rev Med Pharmacol Sci. (2022) 26:6451–58. doi: 10.26355/eurrev_202209_29744 36196695

[33] San Mauro Martín I Garicano Vilar E Romo Orozco DA Mendive Dubourdieu P Paredes Barato V Rincón Barrado M . Hydration status: influence of exercise and diet quality. Am J Lifestyle Med. (2019) 13:414–23. doi: 10.1177/1559827617711906 31285725 PMC6600620

[34] Hakam N Guzman Fuentes JL Nabavizadeh B Sudhakar A Li KD Nicholas C . Outcomes in randomized clinical trials testing changes in daily water intake: a systematic review. JAMA Netw Open. (2024) 7:e2447621. doi: 10.1001/jamanetworkopen.2024.47621 39585691 PMC11589796

[35] Pan B Ge L Lai H Wang Q Wang Q Zhang Q . Association of soft drink and 100% fruit juice consumption with all-cause mortality, cardiovascular diseases mortality, and cancer mortality: a systematic review and dose-response meta-analysis of prospective cohort studies. Crit Rev Food Sci Nutr. (2022) 62:8908–19. doi: 10.1080/10408398.2021.1937040 34121531

