# Supplementary material for: Associations between beverage consumption patterns and adiposity and urinary biomarkers among young adults: a multicenter cross-sectional study in China
Source: Front Nutr. 2026 Jul 24;13:1870358. doi: 10.3389/fnut.2026.1870358 (PMC13449303; doi:10.3389/fnut.2026.1870358)
Supplement: Supplementary file 1 [file Supplementary_file_1.docx]

**Supplementary Materials**

Contents

[Supplementary Methods	2](#_Toc232330239)

[Sample size calculation	2](#_Toc232330240)

[Participants and Recruitment	2](#_Toc232330241)

[Measurement	3](#_Toc232330242)

[Quality Control	4](#_Toc232330243)

[Supplementary Tables	5](#_Toc232330244)

[Supplementary Table 1. Percentage Contribution of Beverage Categories to Total Daily Drinking Fluids Intake by Region and Sex (%).	5](#_Toc232330245)

[Supplementary Table 2. Beverage Consumption Pattern by Region	7](#_Toc232330246)

[Supplementary Table 3. Variance Inflation Factors (VIFs) for the fully adjusted model	8](#_Toc232330247)

[Supplementary Table 4A. Sensitivity Analysis: Associations between beverage intake (per 100 mL increase) and adiposity indices, without adjustment for total energy intake.	9](#_Toc232330248)

[Supplementary Table 4B. Sensitivity Analysis: Associations between beverage intake (per 100 mL increase) and urinary biomarkers, without adjustment for total energy intake.	10](#_Toc232330249)

[Supplementary Table 5. Sensitivity analyses for the association between plain water intake (or daily fluids intake) and adiposity indices.	11](#_Toc232330250)

[Supplementary Table 6A: Sex-specific Interaction Effects in Beverage Consumption on Anthropometric and Blood Pressure Outcomes	12](#_Toc232330251)

[Supplementary Table 6B: Sex-specific Interaction Effects in Beverage Consumption on Renal Function and Biochemical Outcomes	13](#_Toc232330252)

[Supplementary Figures	14](#_Toc232330253)

[Supplementary Figure 1. Flowchart of study	14](#_Toc232330254)

# Supplementary Methods

## Sample size calculation

The minimum required sample size for a cross-sectional design was calculated using the standard formula:

$$n=\frac{Z^{2}\times p\times\left( 1-p \right)}{E^{2}}$$

where *n* is the sample size, *Z* is the Z statistic corresponding to a 95% confidence level (1.96), *p* is the expected prevalence of plain water consumption, and *E* is the acceptable margin of error (0.05). Based on previous national survey data^1^ , approximately 28% of adults met the adequate intake level for plain water (p = 0.28). Accordingly,

$$n=\frac{\left( 1.96 \right)^{2}\times0.28\times\left( 1-0.28 \right)}{\left( 0.05 \right)^{2}}\approx313$$

To account for an expected 20% dropout rate, Thus, a minimum of 322 participants was required for per region; for a total minimum sample size of 2,739. Our actual sample depends on the number of participants actually recruited from each region.

## Participants and Recruitment

A predefined sampling frame was applied to ensure representation from seven geographic regions: Tianjin Medical University (North), Jilin University (Northeast), Shanghai Jiao Tong University (East), Hainan Medical University (South), Yunnan Agricultural University (Southwest), Lanzhou University (Northwest), and Central South University (Central). This regional distribution was intended to capture variation in climate, dietary habits, and sociocultural environments across China. All students provided written informed consent before participating in the study.

Participants were undergraduate and graduate students aged 18–25 years who were in good general health. Individuals were excluded if they smoked, consumed alcohol in excess (>20 g/day), engaged in more than 10 hours per week of vigorous physical activity, had recently taken medications or dietary supplements, or reported a history of chronic illnesses such as gastrointestinal, renal, metabolic, or neurocognitive disorders.

Recruitment was implemented through an integrated online–offline approach. Digital invitations were circulated via popular social media platforms in China (WeChat and QQ), while on-campus promotion relied on posters, information tables, and short announcements in classrooms. All recruitment materials described the study aims, procedures, and eligibility requirements in a neutral manner to minimize self-selection bias.

## Measurement

Anthropometric measurements were obtained following standardized protocols. Standing height and body weight were recorded to the nearest 0.1 cm and 0.1 kg, respectively, using calibrated electronic stadiometers (HDM-300; Huaju, Zhejiang, China) and digital scales (AccuMeasure, Greenwood Village, CO, USA). Body mass index (BMI) was computed as weight in kilograms divided by height squared in meters (kg/m²). Waist circumference (WC) was measured to 0.1 cm precision at the midpoint between the lower margin of the last rib and the iliac crest using a non-elastic measuring tape. Blood pressure was assessed twice in the seated position after a minimum of 5 minutes of rest with an automated sphygmomanometer (Omron HEM-7200; Omron Healthcare, Kyoto, Japan), and the average of the two measurements was used for subsequent analyses.

Potential confounders were prespecified based on conceptual models and prior evidence. Demographic variables included age, sex, ethnicity, and socioeconomic status. The latter was operationalized by city classification into first-tier, new first-tier, second-tier, and third-tier cities according to the Chinese City Tier System, ^2^ which considers economic performance, infrastructure, transportation network, and cultural relevance (National Bureau of Statistics of China).^3^

Lifestyle characteristics were captured through standardized instruments: physical activity was quantified using the International Physical Activity Questionnaire (IPAQ), ^4^ and sleep quality was evaluated with the Pittsburgh Sleep Quality Index (PSQI) ^5^. Dietary variables comprised daily total energy intake, sodium consumption, and macronutrient distribution (protein, carbohydrates, and fat). Sodium intake was further derived from 24-hour urinary sodium excretion multiplied by total urine volume, converted to grams of salt using the following formula:^6^

$$\text{Salt (g)}=\frac{\left[ 24h urinary \text{Na (mmol}\text{/}\text{L)}\times24 h volume\text{ (L)} \right]\times58.42 \left( \text{mg}\text{/}\text{mmol} \right)}{1000 \left( \text{mg}\text{/}\text{g} \right)}$$

Psychological status was assessed using the Self-Rating Anxiety Scale (SAS) ^7^ and Self-Rating Depression Scale (SDS). ^8^ Environmental exposures were characterized by site-specific mean daily temperature and relative humidity, recorded three times per day (10:00, 14:00, and 20:00) with portable sensors (Testo 608-H1; Testo SE, Germany; accuracy ±1 °C, ±0.1% RH) during the study period.

All data collection followed harmonized operating procedures. Demographic and lifestyle data were captured through interviewer-supervised questionnaires to minimize missing information. Dietary intake was validated using duplicate portion sampling: food specimens were frozen at −20 °C, transported under a cold chain, and analyzed centrally. Energy and macronutrient composition were determined according to national standards (GB 28050-2011 for energy and carbohydrate, GB 5009.5-2016 Method 1 for protein, GB 5009.6-2016 Method 2 for fat).

## Quality Control

A comprehensive quality-assurance program was implemented to preserve procedural consistency and ensure data credibility. Study procedures and measurement tools were developed from a systematic review of the literature and consolidated expert input, and their reliability has been demonstrated in prior investigations. A single, detailed operations manual specified all field procedures, and study staff completed centralized training followed by competency certification prior to fieldwork. Trained investigators, using routinely calibrated equipment, performed all anthropometric assessments and biological specimen collection under direct supervision to reduce reliance on self-report.

To maximize the precision of fluid intake estimates, participants completed a validated seven-day fluid diary with volume recording resolution of 5 mL. Water contributed from foods was quantified by the duplicate-portion approach and subsequent moisture determination via direct drying in accordance with national standards. Collected food specimens were frozen at −20 °C, maintained under cold-chain transport, and analyzed in a single core laboratory to minimize between-laboratory variability.

Data handling employed a double-entry workflow with independent cross-checks and automated logical validation to detect missing or inconsistent values, which were queried and rectified promptly. Personal identifiers were removed and replaced with anonymized study codes, and electronic data were stored on encrypted servers with role-based access restrictions. Dedicated on-site quality control teams conducted routine monitoring visits, and any protocol deviations were documented and corrected immediately.

# Supplementary Tables

## Supplementary Table 1. Percentage Contribution of Beverage Categories to Total Daily Drinking Fluids Intake by Region and Sex (%).

| **Region** | **Sex** | **Water** **(%)** | **Coffee and Tea** **(%)** | **100% Fruit Juices** **(%)** | **SSB (%)** | **ASB (%)** | **Alcoholic Beverages** **(%)** | **Other Beverages** **(%)** |
| --- | --- | --- | --- | --- | --- | --- | --- | --- |
| Northeast China (Changchun, CC) | Total | 87.2 | 2.9 | 0.6 | 5.8 | 0 | 0 | 3.6 |
|  | Male | 86.5 | 2.1 | 0.3 | 6.4 | 0 | 0 | 4.7 |
|  | Female | 87.9 | 3.6 | 0.9 | 5.2 | 0 | 0 | 2.5 |
| North China  (Jilin, JL) | Total | 79.4 | 5.5 | 0.1 | 12.9 | 0.6 | 0.6 | 0.9 |
|  | Male | 79.7 | 4.1 | 0 | 13.3 | 1 | 0.9 | 0.9 |
|  | Female | 78.9 | 7.3 | 0.2 | 12.3 | 0.1 | 0.2 | 1.0 |
| Northwest China (Lanzhou, LZ) | Total | 84.2 | 4.7 | 0 | 8.8 | 0 | 0.5 | 1.8 |
|  | Male | 83.4 | 5.2 | 0 | 8.9 | 0 | 0.7 | 1.9 |
|  | Female | 85.1 | 4.2 | 0 | 8.8 | 0 | 0.2 | 1.7 |
| East China  (Shanghai, SH) | Total | 76.4 | 5.6 | 1 | 12.3 | 0.5 | 0.4 | 3.7 |
|  | Male | 77.2 | 3.8 | 0.7 | 13 | 1.1 | 0.9 | 3.2 |
|  | Female | 75.7 | 6.8 | 1.2 | 12.1 | 0.1 | 0.1 | 4 |
| Central China (Changsha, CS) | Total | 79.4 | 5.5 | 0.1 | 12.9 | 0.6 | 0.6 | 0.9 |
|  | Male | 79.7 | 4.1 | 0 | 13.3 | 1 | 0.9 | 0.9 |
|  | Female | 78.9 | 7.3 | 0.2 | 12.3 | 0.1 | 0.2 | 1 |
| Southwest China (Yunnan, YN) | Total | 86.1 | 0.9 | 5.1 | 4.1 | 0 | 0 | 3.7 |
|  | Male | 87.5 | 1.3 | 3.9 | 3.9 | 0 | 0 | 3.3 |
|  | Female | 85.1 | 0.7 | 6 | 4.1 | 0 | 0 | 4.1 |
| South China  (Haikou, HK) | Total | 88.7 | 2.6 | 1.5 | 5.2 | 0.1 | 0 | 2 |
|  | Male | 89.2 | 2.7 | 1.3 | 4.8 | 0.1 | 0 | 1.9 |
|  | Female | 88.1 | 2.5 | 1.7 | 5.6 | 0.1 | 0 | 2 |
| Overall | Total | 83.9 | 3.7 | 1.3 | 8.1 | 0.2 | 0.2 | 2.7 |
|  | Male | 83.6 | 4.1 | 1.6 | 7.8 | 0 | 0.1 | 2.8 |
|  | Female | 84.1 | 3.2 | 0.9 | 8.5 | 0.3 | 0.4 | 2.5 |

Values are expressed as percentages (%) of total daily drinking fluid intake. Regions are grouped by geographic areas with representative cities and abbreviations in parentheses, ordered from north to south. For each region, total population data (Total) are followed by sex-specific data (Male, Female). Beverage categories include plain water, coffee and tea, 100% fruit juices, sugar-sweetened beverages (SSB), artificially sweetened beverages (ASB), alcoholic beverages, and other beverages.

## Supplementary Table 2. Beverage Consumption Pattern by Region

| **Beverage** | **Overall (N=3198)** | **Northeast China (JL, N=499)** | **North China (TJ, N=316)** | **Northwest China (LZ, N=502)** | **East China (SH, N=398)** | **Central China (CS, N=498)** | **Southwest China (YN, N=466)** | **South China (HK, N=519)** | ***p* value** |
| --- | --- | --- | --- | --- | --- | --- | --- | --- | --- |
| Plain water | 1050 (750–1357) | 993 (821–1193) | 1026 (654–1384) | 1101 (815–1538) | 857 (551–1146) | 952 (660–1325) | 920 (600–1363) | 1272 (1117–1423) | <0·001 |
| Coffee and tea | 0 (0–50) | 0 (0–43) | 0 (0–39) | 0 (0–34) | 0 (0–79) | 0 (0–86) | 0 (0–0) | 0 (0–57) | <0·001 |
| 100% fruit juices | 0 (0–0) | 0 (0–0) | 0 (0–0) | 0 (0–0) | 0 (0–0) | 0 (0–0) | 0 (0–0) | 0 (0–26) | <0·001 |
| SSB | 50 (0–150) | 43 (0–86) | 43 (0–152) | 61 (0–193) | 100 (9–206) | 107 (29–229) | 0 (0–0) | 57 (0–102) | <0·001 |
| Other beverages | 0 (0–50) | 29 (0–64) | 21 (0–86) | 0 (0–29) | 0 (0–50) | 0 (0–0) | 0 (0–29) | 0 (0–53) | <0·001 |

Data are presented as median (interquartile range, mL/day). *P* values were calculated using Kruskal–Wallis tests. Median intake of artificially sweetened beverages (ASB) and alcoholic beverages was 0 mL (IQR 0–0) across all regions; however, significant p values reflect differences at the upper tails of the distribution rather than central tendency. Northeast China (JL), North China (TJ), Northwest China (LZ), East China (SH), Central China (CS), Southwest China (YN), South China (HK).

## Supplementary Table 3. Variance Inflation Factors (VIFs) for the fully adjusted model

| Covariates | VIF |
| --- | --- |
| Plain Water (per 100 mL) | 1.11 |
| Gender | 1.27 |
| Age | 1.15 |
| Ethnicity | 1.01 |
| Physical Activity (PA) level | 1.1 |
| SAS scale | 1.26 |
| SDS index | 1.28 |
| Total Energy Intake (kcal/day) | 2.06 |
| Daily salt intake | 1.06 |
| High fat intake | 1.09 |
| High protein intake | 1.56 |
| High carbohydrate intake | 1.37 |
| Geographic Region (Tier) | 1.27 |

VIF, variance inflation factor. All VIF values were well below commonly accepted thresholds for multicollinearity, indicating that multicollinearity was unlikely to materially influence the model estimates.

## Supplementary Table 4A. Sensitivity Analysis: Associations between beverage intake (per 100 mL increase) and adiposity indices, without adjustment for total energy intake.

| Beverage Types | BMI (kg/m²) |  | WC (cm) |  | SBP (mmHg) |  | DBP (mmHg) |  |
| --- | --- | --- | --- | --- | --- | --- | --- | --- |
|  | β [95% CI] | p | β [95% CI] | p | β [95% CI] | p | β [95% CI] | p |
| Plain Water | 0.06 [0.02, 0.10] | 0.007 | 0.14 [0.02, 0.26] | 0.024 | 0.10 [-0.02, 0.23] | 0.110 | 0.01 [-0.08, 0.11] | 0.789 |
| Coffee/Tea | -0.30 [-0.66, 0.06] | 0.103 | -1.12 [-2.16, -0.07] | 0.036 | -0.37 [-1.47, 0.74] | 0.515 | -0.34 [-1.16, 0.49] | 0.423 |
| Fruit Juices | -0.28 [-0.56, 0.01] | 0.055 | -0.91 [-1.74, -0.08] | 0.031 | -0.06 [-0.94, 0.82] | 0.897 | -0.52 [-1.17, 0.14] | 0.120 |
| SSB | 0.11 [-0.02, 0.24] | 0.099 | 1.01 [0.64, 1.38] | <0.001 | 0.24 [-0.15, 0.64] | 0.231 | 0.26 [-0.03, 0.56] | 0.08 |
| ASB | 1.21 [0.31, 2.11] | 0.008 | 5.91 [3.37, 8.46] | <0.001 | 3.39 [0.70, 6.07] | 0.013 | 0.96 [-1.04, 2.96] | 0.345 |
| Alcoholic | -0.30 [-1.27, 0.66] | 0.540 | -0.74 [-3.49, 2.01] | 0.597 | 1.71 [-1.17, 4.59] | 0.243 | 0.64 [-1.49, 2.78] | 0.554 |
| Other Beverages | -0.30 [-0.66, 0.06] | 0.101 | -1.12 [-2.17, -0.08] | 0.035 | -0.37 [-1.47, 0.74] | 0.515 | -0.34 [-1.16, 0.49] | 0.422 |

β (95% CI) represent changes in outcomes per 100 mL/day increase in beverage intake. Models were adjusted for gender, age, ethnicity, PA level, SAS scale, SDS index, daily salt intake, high fat intake, high protein intake, high carbohydrate intake, and geographic region tier. Total energy intake was excluded as part of the sensitivity analysis. p values were derived from mixed-effects models.

## Supplementary Table 4B. Sensitivity Analysis: Associations between beverage intake (per 100 mL increase) and urinary biomarkers, without adjustment for total energy intake.

| Beverage Types | Urinary Cr (μmol/L) |  | Urinary UA (μmol/L) |  | Urinary UA/Cr |  | Urinary Urea |  |
| --- | --- | --- | --- | --- | --- | --- | --- | --- |
|  | β [95% CI] | p | β [95% CI] | p | β [95% CI] | p | β [95% CI] | p |
| Plain Water | -149.58 [-198.98, -100.18] | <0.001 | -10.20 [-17.87, -2.52] | 0.009 | 0.00 [0.00, 0.00] | 0.011 | -3.70 [-4.76, -2.64] | <0.001 |
| Coffee/Tea | 188.36 [-246.06, 622.77] | 0.395 | -6.73 [-73.35, 59.89] | 0.843 | -0.01 [-0.03, 0.01] | 0.230 | 2.02 [-7.31, 11.36] | 0.671 |
| Fruit Juices | 24.12 [-322.64, 370.87] | 0.891 | 1.27 [-51.99, 54.54] | 0.963 | -0.00 [-0.01, 0.01] | 0.972 | -1.94 [-9.38, 5.50] | 0.610 |
| SSB | -23.77 [-180.18, 132.65] | 0.766 | 45.78 [22.04, 69.53] | <0.001 | 0.01 [0.01, 0.02] | <0.001 | -1.68 [-5.04, 1.69] | 0.328 |
| ASB | 476.97 [-589.76, 1543.71] | 0.380 | -42.35 [-205.36, 120.66] | 0.610 | -0.00 [-0.05, 0.04] | 0.935 | 7.76 [-15.22, 30.74] | 0.508 |
| Alcoholic | -204.29 [-1344.87, 936.29] | 0.725 | 19.71 [-154.58, 194.00] | 0.824 | 0.00 [-0.04, 0.05] | 0.875 | 1.77 [-22.80, 26.33] | 0.888 |
| Other Beverages | 188.37 [-246.31, 623.06] | 0.395 | -6.90 [-73.56, 59.75] | 0.839 | -0.01 [-0.03, 0.01] | 0.225 | 2.10 [-7.23, 11.43] | 0.659 |

β (95% CI) represent changes in outcomes per 100 mL/day increase in beverage intake. Models were adjusted for the same covariates as in the primary analyses, except that total energy intake was excluded. p values were derived from mixed-effects models.

## Supplementary Table 5. Sensitivity analyses for the association between plain water intake (or daily fluids intake) and adiposity indices.

| Sensitivity Analysis Model | Outcome | Predictor | β [95% CI] | p |
| --- | --- | --- | --- | --- |
| (a) Exclude Underweight & Obese | BMI (kg/m²) | Plain Water (100 mL) | 0.048 [0.016, 0.081] | 0.004 |
|  | WC (cm) | Plain Water (100 mL) | 0.048 [-0.011, 0.107] | 0.112 |
| (b) Stratified by Sex: Females | BMI (kg/m²) | Plain Water (100 mL) | 0.066 [0.006, 0.127] | 0.032 |
|  | WC (cm) | Plain Water (100 mL) | 0.073 [0.030, 0.116] | <0.001 |
| (b) Stratified by Sex: Males | BMI (kg/m²) | Plain Water (100 mL) | 0.063 [0.024, 0.101] | 0.002 |
|  | WC (cm) | Plain Water (100 mL) | 0.080 [-0.034, 0.194] | 0.167 |
| (c) Predictor: Total Water Intake | BMI (kg/m²) | DFI (100 mL) | 0.130 [-0.026, 0.285] | 0.102 |
|  | WC (cm) | DFI (100 mL) | 0.157 [-0.034, 0.347] | 0.106 |
| (d) Robust Regression | BMI (kg/m²) | Plain Water (100 mL) | 0.243 [0.120, 0.367] | <0.001 |
|  | WC (cm) | Plain Water (100 mL) | 0.144 [0.039, 0.248] | 0.007 |

BMI, Body Mass Index; WC, Waist Circumference; DFI, daily fluids intake. Model (a) excluded participants with BMI < 18.5 or ≥ 28.0 kg/m². Model (d) utilized robust mixed-effects regression to penalize influential outliers.

## Supplementary Table 6A: Sex-specific Interaction Effects in Beverage Consumption on Anthropometric and Blood Pressure Outcomes

| Beverage Types | BMI (kg/m²) |  | WC (cm) |  | SBP (mmHg) |  | DBP (mmHg) |  |
| --- | --- | --- | --- | --- | --- | --- | --- | --- |
|  | β [95% *CI*] | p | β [95% CI] | p | β [95% CI] | p | β [95% CI] | p |
| Plain Water | -0.05 [-0.13, 0.04] | 0.262 | -0.06 [-0.30, 0.18] | 0.625 | -0.15 [-0.40, 0.10] | 0.233 | -0.05 [-0.24, 0.14] | 0.601 |
| WFF | 0.00 [-0.16, 0.16] | 0.989 | -0.17 [-0.62, 0.28] | 0.456 | -0.07 [-0.54, 0.40] | 0.773 | -0.03 [-0.38, 0.32] | 0.873 |
| Coffee/Tea | -0.32 [-1.03, 0.39] | 0.383 | -0.80 [-2.85, 1.25] | 0.442 | -0.32 [-2.47, 1.82] | 0.766 | 0.44 [-1.16, 2.03] | 0.592 |
| Fruit Juices | -0.16 [-1.01, 0.70] | 0.72 | 0.74 [-1.70, 3.18] | 0.551 | 0.14 [-2.41, 2.69] | 0.914 | 1.01 [-0.88, 2.91] | 0.295 |
| SSB | -0.11 [-0.39, 0.17] | 0.433 | -0.15 [-0.93, 0.64] | 0.711 | 0.00 [-0.83, 0.83] | 1 | 0.14 [-0.48, 0.76] | 0.662 |

Sex-specific interactions between beverage consumption and BMI, WC, SBP, and DBP were assessed using multivariable regression models with interaction terms (beverage × sex). Data are presented as β (95% CI), with P values for interaction. Models were adjusted for covariates as described in the Methods. BMI (kg/m²), WC (cm), SBP/DBP (mmHg).

## Supplementary Table 6B: Sex-specific Interaction Effects in Beverage Consumption on Renal Function and Biochemical Outcomes

| Beverage Types | Cr (μmol/L) |  | UA (μmol/L) |  | UA/Cr |  | Urea (μmol/L) |  |
| --- | --- | --- | --- | --- | --- | --- | --- | --- |
|  | *β* [95% *CI*] | *p* | *β* [95% *CI*] | *p* | *β* [95% *CI*] | *p* | *β* [95% *CI*] | *p* |
| Plain Water | -102.91 [-199.22, -6.59] | 0.036 | 7.16 [-7.81, 22.14] | 0.348 | 0.01 [0.00, 0.01] | 0.025 | -2.92 [-4.98, -0.86] | 0.005 |
| WFF | -69.30 [-253.34, 114.73] | 0.460 | 4.44 [-23.80, 32.68] | 0.758 | 0.01 [0.00, 0.01] | 0.188 | -2.73 [-6.70, 1.25] | 0.178 |
| Coffee/Tea | 496.52 [-347.81, 1340.86] | 0.249 | -44.24 [-173.42, 84.95] | 0.502 | -0.01 [-0.05, 0.02] | 0.451 | 6.06 [-12.15, 24.26] | 0.514 |
| Fruit Juices | 867.81 [-143.20, 1878.82] | 0.092 | -80.88 [-235.48, 73.71] | 0.305 | -0.03 [-0.07, 0.02] | 0.203 | 13.47 [-8.33, 35.27] | 0.226 |
| SSB | -116.17 [-443.79, 211.45] | 0.487 | 3.16 [-46.55, 52.87] | 0.901 | 0.00 [-0.01, 0.02] | 0.623 | -3.54 [-10.60, 3.51] | 0.325 |
| ASB | -764.47 [-6223.56, 4694.63] | 0.783 | -351.37 [-1185.32, 482.57] | 0.408 | -0.04 [-0.26, 0.19] | 0.756 | -14.61 [-132.28, 103.06] | 0.808 |
| Alcoholic | 1504.14 [-1225.53, 4233.80] | 0.280 | -122.04 [-539.16, 295.08] | 0.566 | -0.05 [-0.16, 0.06] | 0.401 | 24.06 [-34.80, 82.92] | 0.423 |
| Other | 496.70 [-348.20, 1341.60] | 0.249 | -43.79 [-173.04, 85.46] | 0.506 | -0.01 [-0.05, 0.02] | 0.460 | 5.90 [-12.30, 24.10] | 0.525 |

Sex-specific interactions between beverage consumption and Cr, UA, UA/Cr, and urea were evaluated using multivariable regression models with interaction terms (beverage × sex). Data are presented as β (95% CI), with P values for interaction. Models were adjusted as described in the Methods. All biomarkers are in μmol/L, except UA/Cr.

# Supplementary Figures

## Supplementary Figure 1. Flowchart of study

**Figure 1. Flowchart of participant recruitment, data collection, and analysis**

This flowchart illustrates participant recruitment, data collection, and inclusion in the final analysis. A total of 3,497 healthy university students aged 18–25 years were recruited from seven geographic regions of China. After initial screening, participants completed a 7-day protocol comprising demographic and anthropometric assessments, fluid intake diaries, dietary and lifestyle questionnaires (IPAQ), psychosocial measures (PSQI, SAS, SDS), and environmental monitoring. Following the exclusion of 38 individuals due to non-compliance or incomplete data, 3,198 participants were included in the final analysis of sugar-sweetened beverage (SSB) consumption.

**References:**

1.	Zhang N, Morin C, Guelinckx I, et al. Fluid intake in urban China: results of the 2016 Liq.In (7) national cross-sectional surveys. *Eur J Nutr* 2018; **57**(Suppl 3): 77-88.

2.	China SCo. Notice of the State Council on Adjusting the Criteria for Classifying the Size of Cities. Beijing: State Council of China, 2014.

3.	National Bureau of Statistics of China. China Statistical Yearbook. 2024.

4.	Ekingen T, Sob C, Hartmann C, et al. Associations between hydration status, body composition, sociodemographic and lifestyle factors in the general population: a cross-sectional study. *BMC Public Health* 2022; **22**(1): 900.

5.	Lee KW, Shin D, Song WO. Total Water Intake from Beverages and Foods Is Associated with Energy Intake and Eating Behaviors in Korean Adults. *Nutrients* 2016; **8**(10).

6.	Nowson C, Lim K, Grimes C, et al. Dietary salt intake and discretionary salt use in two general population samples in Australia: 2011 and 2014. *Nutrients* 2015; **7**(12): 10501-12.

7.	Zung WW. A rating instrument for anxiety disorders. *Psychosomatics* 1971; **12**(6): 371-9.

8.	Zung WW. A self-rating depression scale. *Arch Gen Psychiatry* 1965; **12**: 63-70.
